# Physical Activity Behaviors and Negative Safety and Violence Experiences Among High School Students — Youth Risk Behavior Survey, United States, 2023

**DOI:** 10.15585/mmwr.su7304a11

**Published:** 2024-10-10

**Authors:** Kelly Cornett, Shannon L. Michael, Sarah Sliwa, Tiffany J. Chen, Christopher J. Kissler, Izraelle I. McKinnon, Kathleen H. Krause

**Affiliations:** ^1^Division of Adolescent and School Health, National Center for Chronic Disease Prevention and Health Promotion, CDC; ^2^Division of Nutrition, Physical Activity, and Obesity, National Center for Chronic Disease Prevention and Health Promotion, CDC; ^3^Epidemic Intelligence Service, CDC

## Abstract

Schools are in a unique position to offer opportunities for students to be physically active throughout the school day and promote health and well-being. However, experiences that threaten safety or perceptions of safety might affect students’ physical activity behaviors. Using the 2023 national Youth Risk Behavior Survey, six physical activity behaviors and five negative safety and violence experiences were examined from a nationally representative sample of U.S. high school students. This report updates national estimates for physical activity behaviors overall and by sex, grade, race and ethnicity, and sexual identity. In addition, associations between negative experiences and physical activity behaviors were examined, stratified by sex, via unadjusted and adjusted prevalence ratios. Regardless of negative safety and violence experiences, male students had a higher prevalence of meeting aerobic, muscle-strengthening, and both aerobic and muscle-strengthening physical activity guidelines compared with female students. In adjusted models among female students, a positive association was observed between being threatened or injured with a weapon at school and meeting the aerobic guideline, meeting the muscle-strengthening guideline, and playing on ≥1 sports team. Among male students, positive associations were observed between witnessing neighborhood violence and meeting the aerobic guideline and the muscle-strengthening guideline. A negative association was observed between attending physical education classes on all 5 days and witnessing neighborhood violence among female students and being bullied electronically among male students. Physical activity might serve as a mechanism that students employ to cope with negative safety and violence experiences. Understanding current physical activity behaviors among students with these negative experiences will be useful for school leaders, teachers, and public health practitioners who influence physical activity infrastructure and programming in schools and work to support safe, supportive, and inclusive school environments for student health. Although future research is needed to further explore these associations, physical activity continues to be an important behavior to prioritize for adolescent health in the school setting.

## Introduction

Physical activity is important for preventing chronic disease, improving physical and mental health, and improving cognitive functioning (*1*). The Federal *Physical Activity Guidelines for Americans*, second edition, recommends that children and adolescents aged 6–17 years engage in 60 minutes or more of mostly aerobic moderate-to-vigorous physical activity each day (aerobic guideline), as well as muscle-strengthening physical activity on at least 3 days each week (muscle-strengthening guideline) (*1*). In addition, physical activity can serve as a protective factor that promotes the health and emotional well-being of children and adolescents (*2*). Schools are in a unique position to offer opportunities for students to be physically active throughout the school day as part of a Comprehensive School Physical Activity Program (https://www.cdc.gov/healthyschools/physicalactivity/pdf/2019_04_25_PE-PA-Framework_508tagged.pdf) to help meet the youth physical activity guidelines. Lack of physical activity can negatively affect students’ physical and mental health, and have long-term health implications for various health conditions including heart disease, obesity, and type 2 diabetes (*1*). However, even when opportunities for physical activity exist, additional factors, such as students’ actual or perceived lack of safety, might affect physical activity behaviors (*3*). Studies also have demonstrated that consistent sex disparities in students’ physical activity exist (*4*). For example, in 2021, a higher percentage of male than female students met the aerobic and muscle-strengthening guidelines, attended physical education classes on all 5 days, and played on ≥1 sports team (*5*). Furthermore, another study suggests that negative safety and violence experiences for male students might have different associations with physical activity than for female students (*6*). Therefore, it could be important to consider how safety and violence affect participation in physical activity by sex alongside appropriate physical activity infrastructure and programming.

The association between physical activity behavior and negative safety and violence experiences (i.e., experiences of violence and bullying) among students has not been examined using nationally representative data in more than a decade (*6*), and it merits being revisited, especially as concerns around school safety and violence have persisted. CDC’s Youth Risk Behavior Survey Data Summary & Trends Report: 2011–2021 (https://www.cdc.gov/healthyyouth/data/yrbs/pdf/YRBS_Data-Summary-Trends_Report2023_508.pdf) demonstrates that the prevalence of students skipping school due to feeling unsafe has increased and the prevalence of being bullied electronically has not changed (although school-based bullying has declined). Public health and health professionals, state and local health officials, policymakers, and school leaders can use these findings to explore lack of safety as a factor that might influence the implementation of equitable school-based physical activity policies and practices. Understanding current physical activity behaviors among students with these negative experiences will be useful for practitioners who influence physical activity infrastructure and programming in schools and work toward safe, supportive, and inclusive school environments for student health.

## Methods

### Data Source

This report includes data from the 2023 YRBS (N = 20,103), a cross-sectional, school-based survey conducted biennially since 1991. Each survey year, CDC collects data from a nationally representative sample of public and private school students in grades 9–12 in the 50 U.S. states and the District of Columbia. Additional information about YRBS sampling, data collection, response rates, and processing is available in the overview report of this supplement (*7*). The prevalence estimates for physical activity and negative safety and violence experiences for the study population overall and stratified by demographic characteristics are available at https://nccd.cdc.gov/youthonline/App/Default.aspx. The full YRBS questionnaire, data sets, and documentation are available at https://www.cdc.gov/yrbs/index.html. Institutional review boards at CDC and ICF, the survey contractor, approved the protocol for YRBS. Data collection was conducted consistent with applicable Federal law and CDC policy.*

### Measures

Six physical activity behaviors and five negative safety and violence experiences were examined for this report (Table 1). The physical activity behaviors included being physically active for ≥60 minutes per day on all 7 days (i.e., met the Federal youth aerobic guideline), exercising to strengthen or tone muscles on ≥3 days (i.e., met the Federal youth muscle-strengthening guideline), meeting both youth aerobic and muscle-strengthening guidelines, playing on ≥1 sports team, attending physical education classes on all 5 days, and attending physical education classes ≥1 day during an average week.

**TABLE 1:** Questions, response options, and analytic coding for physical activity behavior and negative safety and violence experiences among high school students — Youth Risk Behavior Survey, United States, 2023

Variable	Question	Response option	Analytic coding
Were physically active for a total of ≥60 minutes per day on all 7 days (i.e., met aerobic guideline)	During the past 7 days, on how many days were you physically active for a total of at least 60 minutes per day? (Add up all the time you spent in any kind of physical activity that increased your heart rate and made you breathe hard some of the time.)	0 days, 1 day, 2 days, 3 days, 4 days, 5 days, 6 days, or 7 days	Yes (7 days) versus no (<7 days)
Did exercises to strengthen or tone muscles on ≥3 days (i.e., met muscle-strengthening guideline)	During the past 7 days, on how many days did you do exercises to strengthen or tone your muscles, such as push-ups, sit-ups, or weight lifting?	0 days, 1 day, 2 days, 3 days, 4 days, 5 days, 6 days, or 7 days	Yes (≥3 days) versus no (<3 days)
Met both aerobic and muscle-strengthening guidelines	[See “were physically active for a total of ≥60 minutes per day on all 7 days” and “did exercises to strengthen or tone muscles on ≥3 days.”]	NA	Physically active for ≥60 minutes per day on all 7 days and did exercises to strengthen or tone muscles on ≥3 days versus physically active for ≥60 minutes per day on <7 days or did exercises to strengthen or tone muscles on <3 days
Played on ≥1 sports team	During the past 12 months, on how many sports teams did you play? (Count any teams run by your school or community groups.)	0 teams, 1 team, 2 teams, or ≥3 teams	Yes (≥1 team) versus no (0 teams)
Attended physical education classes on all 5 days	In an average week when you are in school, on how many days do you go to physical education (PE) classes?	0 days, 1 day, 2 days, 3 days, 4 days, or 5 days	Yes (5 days) versus no (<5 days)
Attended physical education classes on ≥1 day			Yes (≥1 day) versus no (0 day)
Skipped school due to feeling unsafe	During the past 30 days, on how many days did you not go to school because you felt you would be unsafe at school or on your way to or from school?	0 days, 1 day, 2 or 3 days, 4 or 5 days, or ≥6 days	Yes (≥ 1 day) versus no (0 days)
Threatened or injured on school property	During the past 12 months, how many times has someone threatened or injured you with a weapon such as a gun, knife, or club on school property?	0 times, 1 time, 2 or 3 times, 4 or 5 times, 6 or 7 times, 8 or 9 times, 10 or 11 times, or ≥12 times	Yes (≥ 1 time) versus no (0 time)
Witnessed neighborhood violence	Have you ever seen someone get physically attacked, beaten, stabbed, or shot in your neighborhood?	Yes or no	Yes versus no
Bullied at school	During the past 12 months, have you ever been bullied on school property?	Yes or no	Yes versus no
Bullied electronically	During the past 12 months, have you ever been electronically bullied? (Count being bullied through texting, Instagram, Facebook, or other social media.)	Yes or no	Yes versus no

**Abbreviation:** NA = not applicable.

The negative safety and violence experiences included skipping school due to feeling unsafe, being threatened or injured with a weapon on school property, witnessing neighborhood violence, being bullied at school, and being bullied electronically. Demographic variables included sex (female and male), grade (9, 10, 11, and 12), sexual identity (heterosexual; lesbian, gay, or bisexual; questioning [I am not sure about my sexual identity/questioning] or describe identity in some other way [I describe my identity some other way]), and race and ethnicity (American Indian or Alaska Native [AI/AN], Asian, Black or African American [Black], Native Hawaiian or other Pacific Islander [NH/OPI], White, Hispanic or Latino [Hispanic], or multiracial [selected >1 racial category]. (Persons of Hispanic or Latino origin might be of any race but are categorized as Hispanic; all racial groups are non-Hispanic.)

### Analysis

Prevalence and 95% CIs for each physical activity behavior were estimated, overall and by demographic characteristics. Differences between demographic subgroups were determined by pairwise *t*-test analyses. In addition, the prevalence and 95% CIs of each physical activity behavior were estimated, overall and stratified by sex, among students who did and did not have negative safety and violence experiences. Sex-stratified prevalence ratios (PRs) and adjusted PRs (aPRs) were calculated to assess the association between each physical activity behavior and each experience (adjusted for grade, race and ethnicity, and sexual identity). All prevalence estimates and measures of association were determined using Taylor series linearization. PRs and aPRs were calculated using logistic regression with predicted marginals. PRs were considered statistically significant if 95% CIs did not include a value of 1.0 or p<0.05. All analyses were conducted in SAS-callable SUDAAN (version 11.0.3; RTI International) using sample weights to account for complex survey design.

## Results

### Physical Activity Behaviors

Overall, approximately half of high school students played on ≥1 sports team (51.9%), exercised to strengthen or tone their muscles on ≥3 days per week (met the muscle-strengthening guideline) (51.1%), and attended physical education classes at least 1 day a week (49.9%) (Table 2). Approximately one fourth of students were physically active for ≥60 minutes per day on all 7 days (met the aerobic guideline) (24.6%), and fewer met both the aerobic and muscle-strengthening guidelines (16.5%). Male students had a higher prevalence of engaging in all physical activity behaviors compared with female students. The prevalence of four physical activity behaviors (met the aerobic guideline, met the muscle-strengthening guideline, met both guidelines, and played on ≥1 sports team) were lower among Asian, Black, and Hispanic students compared with White students. The prevalence of all physical activity behaviors was lower among students in grade 12 compared with students in grade 9, and among lesbian, gay, or bisexual students, and students who are questioning their identity or who identify in some other way compared with heterosexual students.

**TABLE 2:** Prevalence of high school students who reported physical activity behaviors, by demographic characteristics — Youth Risk Behavior Survey, United States, 2023*

Characteristic	Were physically active for a total of ≥60 minutes per day on all 7 days (met aerobic guideline) % (95% CI)^†^	Did exercises to strengthen or tone muscles on ≥3 days (met muscle-strengthening guideline) % (95% CI)^†^	Met both aerobic and muscle-strengthening guidelines % (95% CI)^†^	Played on ≥1 sports team % (95% CI)^†^	Went to physical education classes on all 5 days % (95% CI)^†^	Went to physical education classes on ≥1 day % (95% CI)^†^
**Sex**						
Female	16.6 (15.0–18.3)^§^	37.2 (34.3–40.2)^§^	9.4 (7.7–11.3)^§^	48.1 (45.0–51.2)^§^	23.1 (18.3–28.9)^§^	44.6 (39.2–50.2)^§^
Male	32.2 (30.0–34.4)	63.8 (60.7–66.8)	23.5 (20.2–27.1)	55.7 (52.0–59.4)	31.4 (24.9–38.6)	54.7 (49.4–59.8)
**Race and ethnicity** ^¶^						
American Indian or Alaska Native	31.9 (14.5–56.4)	44.2 (31.2–58.1)	14.3 (9.1–21.9)**	57.0 (44.7–68.4)	34.9 (23.0–49.0)	53.1 (39.9–65.8)
Asian	15.6 (11.6–20.7)^††,§§,¶¶,^***	45.6 (39.5–51.7)^§§,^***	9.1 (6.3–12.9)^§§,¶¶,^***^,†††^	46.0 (37.5–54.8)^§§,^***	20.6 (12.8–31.3)^§§,†††^	53.2 (38.6–67.3)
Black or African American	21.5 (19.3–24.0)^§§§,¶¶¶^	46.7 (42.5–51.0)***^,§§§^	12.2 (9.2–16.0)^§§§,¶¶¶,^****	49.8 (45.7–53.9)^§§§,¶¶¶^	24.4 (17.2–33.3)	48.1 (39.9–56.3)
Native Hawaiian or other Pacific Islander	29.3 (19.1–42.1)	52.6 (39.1–65.7)	27.3 (17.9–39.1)	56.6 (44.6–67.9)	35.2 (19.8–54.5)	55.7 (38.9–71.3)
White	29.0 (26.7–31.4)	54.3 (49.5–59.0)	19.5 (16.1–23.3)	56.2 (52.0–60.3)	26.2 (20.5–33.0)	48.4 (42.7–54.2)
Hispanic or Latino	20.1 (17.3–23.3)^††††,§§§§^	48.8 (45.2–52.4)^††††^	14.4 (11.1–18.4)^††††,§§§§,¶¶¶¶^	47.3 (43.6–51.0)^††††,§§§§^	30.9 (23.4–39.7)	51.9 (45.3–58.4)
Multiracial	26.3 (21.6–31.5)	56.2 (49.8–62.5)	20.4 (16.1–25.5)	57.0 (51.2–62.5)	28.9 (20.9–38.6)	50.6 (42.7–58.5)
**Grade**						
9	26.9 (24.4–29.6)*****	55.3 (51.5–59.1)*****^,†††††^	17.5 (14.4–20.9)	57.2 (53.1–61.2)*****^,†††††^	39.5 (30.5–49.3)*****^,†††††,§§§§§^	71.1 (64.1–77.2)*****^,†††††,§§§§§^
10	26.4 (23.5–29.6)^¶¶¶¶¶^	52.1 (47.5–56.6)	18.2 (14.6–22.4)^¶¶¶¶¶^	54.2 (50.2–58.2)^¶¶¶¶¶^	30.3 (23.5–38.1)^¶¶¶¶¶,^******	53.3 (47.1–59.4)^¶¶¶¶¶,^******
11	23.7 (21.1–26.4)	48.8 (44.0–53.6)	16.2 (13.0–19.9)	50.2 (45.9–54.5)	21.4 (16.2–27.7)^††††††^	40.2 (33.3–47.6)^††††††^
12	21.0 (18.3–24.1)	47.9 (44.0–51.7)	14.0 (11.3–17.2)	46.1 (41.1–51.3)	17.1 (12.9–22.3)	33.0 (26.2–40.6)
**Sexual identity**						
Heterosexual	28.8 (26.9–30.7)^§§§§§§,¶¶¶¶¶¶^	57.7 (54.5–60.9)^§§§§§§,¶¶¶¶¶¶^	20.3 (17.4–23.4)^§§§§§§,¶¶¶¶¶¶^	57.9 (54.5–61.1)^§§§§§§,¶¶¶¶¶¶^	29.0 (23.0–35.9)^§§§§§§,¶¶¶¶¶¶^	52.2 (46.5–57.8)^§§§§§§,¶¶¶¶¶¶^
Lesbian, gay, or bisexual	12.7 (10.7–15.0)	31.7 (27.8–35.9)	6.9 (5.2–9.2)	37.7 (34.1–41.4)	20.9 (16.2–26.5)	41.7 (36.6–47.0)
Described identity in some other way or questioning	12.7 (10.5–15.4)	27.4 (23.5–31.7)	5.5 (3.6–8.3)	34.0 (29.3–39.0)	23.4 (17.7–30.2)	43.8 (37.8–49.9)
**Total**	**24.6 (22.8–26.5)**	**51.1 (48.0–54.1)**	**16.5 (14.0–19.3)**	**51.9 (48.9–54.9)**	**27.4 (21.9–33.6)**	**49.9 (44.8–55.0)**

* N = 20,103 respondents. The total number of students answering each question varied. Data might be missing because 1) the question did not appear in that student’s questionnaire, 2) the student did not answer the question, or 3) the response was set to missing because of an out-of-range response or logical inconsistency. Percentages in each category are calculated on the known data.

^†^ Weighted prevalence and corresponding 95% CI.

^§^ Female students significantly differed from male students, based on *t*-test analysis with Taylor series linearization (p<0.05).

^¶^ Persons of Hispanic or Latino origin might be of any race but are categorized as Hispanic; all racial groups are non-Hispanic.

** American Indian or Alaska Native students significantly differed from Native Hawaiian or other Pacific Islander students, based on *t*-test analysis with Taylor series linearization (p<0.05).

^††^ Asian students significantly differed from Black or African American students, based on *t*-test analysis with Taylor series linearization (p<0.05).

^§§^ Asian students significantly differed from multiracial students, based on *t*-test analysis with Taylor series linearization (p<0.05).

^¶¶^ Asian students significantly differed from Native Hawaiian or other Pacific Islander students, based on *t*-test analysis with Taylor series linearization (p<0.05).

*** Asian students significantly differed from White students, based on *t*-test analysis with Taylor series linearization (p<0.05).

^†††^ Asian students significantly differed from Hispanic or Latino students, based on *t*-test analysis with Taylor series linearization (p<0.05).

^§§§^ Black or African American students significantly differed from multiracial students, based on *t*-test analysis with Taylor series linearization (p<0.05).

^¶¶¶^ Black or African American students significantly differed from White students, based on *t*-test analysis with Taylor series linearization (p<0.05).

**** Black or African American students significantly differed from Native Hawaiian or other Pacific Islander, based on *t*-test analysis with Taylor series linearization (p<0.05).

^††††^ Hispanic or Latino students significantly differed from multiracial students, based on *t*-test analysis with Taylor series linearization (p<0.05).

^§§§§^ Hispanic or Latino students significantly differed from White students, based on *t*-test analysis with Taylor series linearization (p<0.05).

^¶¶¶¶^ Hispanic or Latino students significantly differed from Native Hawaiian or other Pacific Islander students, based on *t*-test analysis with Taylor series linearization (p<0.05).

***** Students in grade 9 significantly differed from students in grade 12, based on *t*-test analysis with Taylor series linearization (p<0.05).

^†††††^ Students in grade 9 significantly differed from students in grade 11, based on *t*-test analysis with Taylor series linearization (p<0.05).

^§§§§§^ Students in grade 9 significantly differed from students in grade 10, based on *t*-test analysis with Taylor series linearization (p<0.05).

^¶¶¶¶¶^ Students in grade 10 significantly differed from students in grade 12, based on *t*-test analysis with Taylor series linearization (p<0.05).

****** Students in grade 10 significantly differed from students in grade 11, based on *t*-test analysis with Taylor series linearization (p<0.05).

^††††††^ Students in grade 11 significantly differed from students in grade 12, based on *t*-test analysis with Taylor series linearization (p<0.05).

^§§§§§§^ Heterosexual students significantly differed from lesbian, gay, or bisexual students, based on *t*-test analysis with Taylor series linearization (p<0.05).

^¶¶¶¶¶¶^ Heterosexual students significantly differed from students who described identity in some other way or were questioning, based on *t*-test with Taylor series linearization (p<0.05).

### Physical Activity Behaviors by Negative Safety and Violence Experiences

Overall, students who skipped school due to feeling unsafe had a lower prevalence of meeting the aerobic guideline compared with students who did not skip school (Tables 3 and 4). Students who were bullied electronically had a lower prevalence of meeting the muscle-strengthening guideline and attending physical education class on all 5 days compared with students who were not bullied electronically. Conversely, students who were threatened or injured with a weapon at school had a higher prevalence of meeting the muscle-strengthening guideline and playing on ≥1 sports team compared with students who were not threatened or injured with a weapon at school.

**TABLE 3:** Prevalence of physical activity behaviors among high school students, by sex and negative safety and violence experiences — Youth Risk Behavior Survey, United States, 2023*

Negative safety and violence experiences	Were physically active for a total of ≥60 minutes per day on all 7 days (met aerobic guideline)			Did exercises to strengthen or tone muscles on ≥3 days (met muscle-strengthening guideline)			Met both aerobic and muscle-strengthening guidelines		
	Overall % (95% Cl)	Female % (95% Cl)	Male % (95% Cl)	Overall % (95% Cl)	Female % (95% Cl)	Male % (95% Cl)	Overall % (95% Cl)	Female % (95% Cl)	Male % (95% Cl)
Skipped school due to feeling unsafe									
Yes	**20.7 (17.6–24.1)^†^**	14.8 (11.5–18.9)	29.8 (23.6–36.8)	**48.5 (45.5–51.4**)	38.4 (35.4–41.5)	63.8 (57.5–69.6)	**14.0 (11.1–17.6)**	9.9 (7.3–13.4)	20.6 (14.3–28.7)
No	**25.3 (23.3–27.3)**	16.9 (15.2–18.8)	32.5 (30.3–34.7)	**51.4 (48.1–54.7)**	37.0 (33.7–40.4)	63.8 (60.6–66.9)	**16.9 (14.2–20.0)**	9.3 (7.5–11.4)	23.8 (20.5–27.5)
Threatened or injured with a weapon on school property									
Yes	**26.4 (23.0–30.1)**	20.5 (16.3–25.4)	31.7 (27.9–35.7)	**60.6 (55.7–65.4)^§^**	48.6 (42.9–54.2)^§^	71.7 (64.4–78.0)^§^	**16.5 (13.0–20.7)**	12.2 (8.5–17.0)	20.4 (16.0–25.6)
No	**24.5 (22.5–26.5)**	16.3 (14.5–18.2)	32.2 (29.8–34.6)	**50.1 (46.9–53.4)**	36.3 (33.3–39.3)	63.1 (59.7–66.4)	**16.6 (14.0–19.7)**	9.1 (7.4–11.2)	24.0 (20.5–27.8)
Witnessed neighborhood violence									
Yes	**26.5 (23.1–30.1)**	15.7 (12.9–19.0)	35.0 (30.6–39.7)	**53.5 (49.6–57.3)**	36.1 (31.4–41.0)	67.3 (62.7–71.6)^¶^	**20.3 (16.8–24.3)**	10.3 (7.7–13.6)	28.4 (23.7–33.5)
No	**25.0 (23.3–26.8)**	17.3 (15.4–19.5)	32.3 (30.1–34.5)	**50.5 (46.8–54.2)**	37.7 (34.2–41.3)	62.6 (58.9–66.2)	**19.1 (17.0–21.4)**	11.3 (9.5–13.3)	26.6 (24.1–29.4)
Bullied at school									
Yes	**23.0 (19.9–26.4)**	19.1 (16.3–22.3)	27.6 (22.7–33.1)**	**50.4 (46.5–54.4)**	40.9 (37.1–44.8)**	62.5 (57.4–67.3)	**16.1 (13.0–19.8)**	12.6 (10.0–15.8)**	20.3 (15.8–25.7)
No	**25.2 (23.3–27.1)**	16.0 (14.1–18.1)	33.1 (31.0–35.3)	**51.4 (48.1–54.6)**	36.4 (33.2–39.7)	64.2 (60.9–67.3)	**16.7 (14.1–19.7)**	8.5 (6.8–10.6)	24.2 (20.8–27.8)
Bullied electronically									
Yes	**22.3 (18.2–27.1)**	18.6 (15.1–22.7)	28.4 (22.1–35.7)	**47.8 (43.5–52.2)^††^**	40.3 (36.2–44.6)	60.8 (54.1–67.0)	**14.5 (10.8–19.2)**	11.0 (8.2–14.7)	20.1 (14.2–27.6)
No	**25.2 (23.3–27.1)**	16.2 (14.4–18.1)	32.7 (30.6–34.8)	**51.7 (48.6–54.8)**	36.5 (33.3–39.9)	64.2 (61.2–67.2)	**17.0 (14.4–19.8)**	8.9(7.2–11.0)	23.9 (20.8–27.4)

* N = 20,103 respondents. The total number of students answering each question varied. Data might be missing because 1) the question did not appear in that student’s questionnaire, 2) the student did not answer the question, or 3) the response was set to missing because of an out-of-range response or logical inconsistency. Percentages in each category are calculated on the known data.

^†^ Students who skipped school due to feeling unsafe significantly different from students who did not skip school, based on *t*-test analysis with Taylor series linearization (p<0.05).

^§^ Students who were threatened or injured with a weapon on school property significantly differed from students who were not threatened or injured with a weapon on school property, based on *t*-test analysis with Taylor series linearization (p<0.05).

^¶^ Students who witnessed neighborhood violence significantly differed from students who did not witness neighborhood violence, based on *t*-test analysis with Taylor series linearization (p<0.05).

** Students who were bullied at school significantly differed from students who were not bullied at school, based on *t*-test analysis with Taylor series linearization (p<0.05).

^††^ Students who were bullied electronically significantly differed from students who were not bullied electronically, based on *t*-test analysis with Taylor series linearization (p<0.05).

**TABLE 4:** Prevalence of physical activity behaviors among high school students, by sex and negative safety and violence experiences — Youth Risk Behavior Survey, United States, 2023*

Negative safety and violence experiences	Played on ≥1 sports team			Went to physical education classes on all 5 days			Went to physical education classes on ≥1 day		
	Overall % (95% Cl)	Female % (95% Cl)	Male % (95% Cl)	Overall % (95% Cl)	Female % (95% Cl)	Male % (95% Cl)	Overall % (95% Cl)	Female % (95% Cl)	Male % (95% Cl)
Skipped school due to feeling unsafe									
Yes	**50.4 (46.5–54.3)**	46.4 (40.7–52.1)	57.2 (49.1–64.9)	**27.4 (21.3–34.5)**	23.4 (18.4–29.3)	34.2 (24.9–44.9)	**50.4 (43.8–57.1)**	45.7 (38.8–52.7)	58.1 (49.2–66.5)
No	**52.3 (49.2–55.3)**	48.6 (45.2–52.0)	55.6 (52.0–59.3)	**27.4 (21.7–33.9)**	23.1 (18.0–29.2)	31.0 (24.5–38.4)	**49.8 (44.7–55.0)**	44.5 (38.9–50.2)	54.2 (49.1–59.3)
Threatened or injured with a weapon on school property									
Yes	**58.2 (54.1–62.3)^†^**	56.9 (49.9–63.6)^†^	60.5 (54.1–66.5)	**29.8 (23.4–37.2)**	23.6 (17.4–31.2)	36.0 (27.6–45.4)	**52.3 (47.3–57.2)**	43.6 (36.4–51.0)	59.4 (53.0–65.5)
No	**51.2 (47.9–54.5)**	46.9 (43.3–50.5)	55.4 (51.5–59.3)	**27.6 (22.1–33.9)**	23.4 (18.4–29.3)	31.5 (25.0–38.7)	**50.1 (44.9–55.4)**	45.3 (39.6–51.1)	54.7 (49.3–60.0)
Witnessed neighborhood violence									
Yes	**52.6 (48.2–57.0)**	46.7 (41.7–51.8)	57.4 (51.4–63.1)	**29.9 (24.6–35.8)**	22.0 (16.5–28.7)	36.8 (31.1–42.9)	**52.3 (46.6–57.9)**	44.0 (36.8–51.5)	58.8 (53.0–64.4)
No	**52.2 (48.8–55.6)**	48.5 (45.0–52.1)	56.0 (51.8–60.1)	**29.5 (24.1–35.7)**	26.0 (21.0–31.6)	32.8 (26.1–40.3)	**51.8 (46.0–57.5)**	47.0 (40.7–53.4)	56.3 (50.5–62.0)
Bullied at school									
Yes	**51.8 (48.0–55.6)**	50.6 (46.1–55.2)	53.4 (47.6–59.0)	**26.5 (20.8–33.0)**	23.3 (18.4–29.0)	30.3 (22.7–39.2)	**51.2 (45.8–56.5)**	47.0 (40.9–53.3)	56.0 (49.9–62.0)
No	**52.2 (48.9–55.4)**	47.5 (44.3–50.7)	56.3 (52.3–60.3)	**27.5 (21.8–33.9)**	22.8 (17.6–29.1)	31.5 (25.1–38.7)	**49.3 (44.0–54.7)**	43.6 (37.7–49.7)	54.3 (49.0–59.5)
Bullied electronically									
Yes	**50.8 (46.5–55.1)**	51.5 (46.5–56.6)	50.2 (43.1–57.3)	**22.8 (16.8–30.3)^§^**	21.6 (16.0–28.5)	25.1 (17.3–34.9)^§^	**47.8 (42.2–53.4)**	45.8 (39.7–52.0)	50.6 (44.2–56.9)
No	**52.2 (49.0–55.4)**	47.2 (44.1–50.4)	56.6 (52.8–60.3)	**28.3 (22.6–34.8)**	23.6 (18.3–29.8)	32.3 (25.8–39.5)	**50.2 (44.9–55.4)**	44.2 (38.4–50.1)	55.1 (49.8–60.4)

* N = 20,103 respondents. The total number of students answering each question varied. Data might be missing because 1) the question did not appear in that student’s questionnaire, 2) the student did not answer the question, or 3) the response was set to missing because of an out-of-range response or logical inconsistency. Percentages in each category are calculated on the known data.

^†^ Students who were threatened or injured with a weapon on school property significantly differed from students who were not threatened or injured with a weapon on school property, based on *t*-test with Taylor series linearization (p<0.05).

^§^ Students who were bullied electronically significantly differed from students who were not bullied electronically, based on *t*-test with Taylor series linearization (p<0.05).

Among male and female students, the prevalence of physical activity behavior differed by negative safety and violence experience. For example, the prevalence of meeting the muscle-strengthening guideline was higher among female students who were threatened or injured with a weapon at school (48.6%) or bullied at school (40.9%) compared with female students without these experiences (36.3% and 36.4%, respectively). Male students who were threatened or injured with a weapon at school had a higher prevalence (71.7%) of meeting the muscle-strengthening guideline compared with male students without this negative experience (63.1%). Male students who were bullied at school had a lower prevalence of meeting the aerobic guideline (27.6%) and male students who were bullied electronically had a lower prevalence of attending physical education class on all 5 days (25.1%) compared with their male peers who did not experience these kinds of bullying (33.1% and 32.3%, respectively).

### Associations Between Physical Activity Behaviors and Negative Safety and Violence Experiences by Sex

In adjusted models, among female students, a positive association was observed between being threatened or injured with a weapon at school and meeting the aerobic guideline, meeting the muscle-strengthening guideline, and playing on ≥1 sports teams (Tables 5 and 6). In addition, among female students, there was a positive association between being bullied at school and meeting the aerobic guideline and meeting both aerobic and muscle-strengthening guidelines.

**TABLE 5:** Associations between negative safety and violence experiences and physical activity behaviors among high school students, by sex — Youth Risk Behavior Survey, United States, 2023*

Negative safety and violence experience^†^	Were physically active for a total of ≥60 minutes per day on all 7 days (met aerobic guideline)				Did exercises to strengthen or tone muscles on ≥3 days (met muscle-strengthening guideline)				Met both aerobic and muscle-strengthening guidelines			
	Female		Male		Female		Male		Female		Male	
	PR^§^ (95% CI)	aPR^¶^ (95% CI)	PR^§^ (95% CI)	aPR^¶^ (95% CI)	PR^§^ (95% CI)	aPR^¶^ (95% CI)	PR^§^ (95% CI)	aPR^¶^ (95% CI)	PR^§^ (95% CI)	aPR^¶^ (95% CI)	PR^§^ (95% CI)	aPR^¶^ (95% CI)
Skipped school due to feeling unsafe	0.88 (0.68–1.14)	0.98 (0.77–1.26)	0.92 (0.74–1.15)	1.04 (0.84–1.28)	1.04 (0.93–1.15)	1.06 (0.96–1.16)	1.00 (0.91–1.10)	1.07 (0.99–1.16)	1.07 (0.76–1.51)	1.19 (0.86–1.65)	0.86 (0.61– 1.22)	1.01 (0.73– 1.41)
Threatened or injured with a weapon on school property	1.26 (0.98–1.62)	1.34 (1.03–1.74)**	0.99 (0.86–1.13)	1.12 (0.97–1.29)	1.34 (1.20–1.49)**	1.28 (1.12–1.45)**	1.14 (1.03–1.25)**	1.17 (1.07–1.28)**	1.33 (0.93–1.91)	1.38 (0.98–1.96)	0.85 (0.70– 1.03)	0.98 (0.82–1.17)
Witnessed neighborhood violence	0.91 (0.73–1.13)	1.00 (0.80–1.26)	1.08 (0.94–1.24)	1.17 (1.02–1.33)**	0.96 (0.81–1.12)	1.01 (0.88–1.16)	1.07 (1.00–1.15)**	1.11 (1.04–1.18)**	0.91 (0.67–1.22)	1.03 (0.76–1.38)	1.07 (0.91– 1.25)	1.15 (0.99–1.34)
Bullied at school	1.19 (0.96–1.47)	1.21 (1.01–1.45)**	0.83 (0.69–1.01)	0.91 (0.78–1.06)	1.12 (1.01–1.25)**	1.09 (0.99–1.20)	0.97 (0.90–1.05)	1.02 (0.93–1.11)	1.48 (1.13– 1.94)**	1.53 (1.22– 1.93)**	0.84 (0.68–1.03)	0.93 (0.78– 1.12)
Bullied electronically	1.15 (0.90–1.47)	1.19 (0.98–1.45)	0.87 (0.68–1.10)	1.02 (0.88–1.19)	1.10 (0.97–1.25)	1.05 (0.93–1.18)	0.95 (0.86–1.04)	0.98 (0.90–1.07)	1.23 (0.90–1.69)	1.28 (0.98– 1.67)	0.84 (0.63–1.11)	1.01 (0.85–1.20)

**Abbreviations:** aPR = adjusted prevalence ratio; PR = prevalence ratio.

* N = 20,103 respondents. The total number of students answering each question varied. Data might be missing because 1) the question did not appear in that student’s questionnaire, 2) the student did not answer the question, or 3) the response was set to missing because of an out-of-range response or logical inconsistency. Percentages in each category are calculated on the known data.

^†^ Each negative safety experience was included in a separate model with each of the physical activity behaviors, with no other negative safety experiences adjusted for in any model.

^§^ PR for physical activity behaviors, comparing students with negative safety experiences to those without (referent group).

^¶^ aPR for physical activity behaviors, adjusted for grade, race and ethnicity, and sexual identity, comparing students with negative safety experiences to those without (referent group).

** Estimates were considered statistically significant if the 95% Cis did not include 1.0. Certain 95% CIs include 1.0 because of rounding.

**TABLE 6:** Associations between negative safety and violence experiences and physical activity behaviors among high school students, by sex — Youth Risk Behavior Survey, United States, 2023*

Negative safety and violence experience^†^	Played on ≥1 sports team				Went to physical education classes on all 5 days				Went to physical education classes on ≥1 day			
	Female		Male		Female		Male		Female		Male	
	PR^§ ^(95% CI)	aPR^¶ ^(95% CI)	PR^§ ^(95%CI)	aPR^¶ ^(95% CI)	PR^§ ^(95%CI)	aPR^¶ ^(95% CI)	PR^§ ^(95% CI)	aPR^¶ ^(95% CI)	PR^§ ^(95% CI)	aPR^¶ ^(95% CI)	PR^§ ^(95% CI)	aPR^¶ ^(95% CI)
Skipped school due to feeling unsafe	0.95 (0.83–1.09)	0.98 (0.84–1.13)	1.03 (0.91–1.17)	1.10 (0.98–1.22)	1.01 (0.82–1.24)	0.99 (0.79–1.24)	1.10 (0.84–1.44)	1.10 (0.84–1.45)	1.03 (0.91–1.16)	1.00 (0.89–1.13)	1.07 (0.95–1.21)	1.06 (0.94–1.19)
Threatened or injured with a weapon on school property	1.21 (1.06–1.39)**	1.22 (1.06–1.40)**	1.09 (0.99–1.20)	1.15 (1.05–1.26)**	1.01 (0.79–1.29)	0.95 (0.75–1.21)	1.14 (0.95–1.38)	1.06 (0.87–1.30)	0.96 (0.82–1.13)	0.89 (0.75–1.05)	1.09 (0.98–1.21)	0.99 (0.89–1.10)
Witnessed neighborhood violence	0.96 (0.86–1.08)	1.04 (0.94–1.16)	1.02 (0.93–1.13)	1.05 (0.96–1.15)	0.85 (0.69–1.04)	0.78 (0.64–0.95)**	1.12 (0.96–1.31)	1.09 (0.96–1.25)	0.94 (0.83–1.05)	0.90 (0.80–1.01)	1.04 (0.97–1.12)	1.05 (0.98–1.12)
Bullied at school	1.07 (0.98–1.17)	1.03 (0.94–1.12)	0.95 (0.85–1.05)	1.01 (0.91–1.12)	1.02 (0.82–1.26)	0.98 (0.80–1.20)	0.96 (0.82–1.13)	0.96 (0.85–1.10)	1.08 (0.96–1.21)	1.01 (0.91–1.12)	1.03 (0.96–1.11)	1.00 (0.94–1.07)
Bullied electronically	1.09 (0.98–1.21)	1.05 (0.95–1.17)	0.89 (0.78–1.01)	0.96 (0.85–1.09)	0.92 (0.70–1.20)	0.93 (0.74–1.16)	0.78 (0.60–1.00)	0.86 (0.76–0.98)**	1.04 (0.93–1.15)	0.98 (0.89–1.09)	0.92 (0.83–1.01)	0.93 (0.86–1.01)

**Abbreviations:** aPR = adjusted prevalence ratio; PR = prevalence ratio.

* N = 20,103 respondents. The total number of students answering each question varied. Data might be missing because 1) the question did not appear in that student’s questionnaire, 2) the student did not answer the question, or 3) the response was set to missing because of an out-of-range response or logical inconsistency. Percentages in each category are calculated on the known data.

^†^ Each negative safety experience was included in a separate model with each of the physical activity behaviors, with no other negative safety experiences adjusted for in any model.

^§^ PR for physical activity behaviors, comparing students with negative safety experiences to those without (referent group).

^¶^ aPR for physical activity behaviors, adjusted for grade, race and ethnicity, and sexual identity, comparing students with negative safety experiences to those without (referent group).

** Estimates were considered statistically significant if the 95% CIs did not include 1.0. Some 95% CIs include 1.0 because of rounding.

Among male students, there was a positive association between being threatened or injured with a weapon at school and meeting the muscle-strengthening guideline and playing on ≥1 sports team. In addition, among male students, a positive association between witnessing neighborhood violence and meeting the aerobic guideline and meeting the muscle-strengthening guideline was observed.

Negative associations were observed between the negative safety and violence experiences and physical education attendance. In adjusted models, among female students, a negative association was observed between witnessing neighborhood violence and attending physical education classes on all 5 days. In adjusted models, among male students, a negative association was observed between being bullied electronically and attending physical education classes on all 5 days.

## Discussion

Although nationwide efforts to promote physical activity among adolescents exist, the findings of this report indicate that >80% of U.S. high school students are not meeting the nationally recommended aerobic and muscle-strengthening guidelines for physical activity. In addition, disparities were observed in meeting guidelines, whereby male students, heterosexual students, and White students were more likely to meet the physical activity guidelines. This report found, for male and female students, that certain negative safety and violence experiences were associated with a higher prevalence of engagement in physical activity behaviors and that associations varied by sex. In adjusted models, a positive association was observed between being threatened or injured on school property and meeting the muscle-strengthening guideline, among both male and female students. In contrast, being bullied at school was positively associated with meeting the aerobic guideline and meeting both the aerobic and muscle-strengthening guidelines among female but not male students, in adjusted models.

Previous studies have demonstrated the role of physical activity as a protective factor that promotes students’ health and well-being (*8*). Daily physical activity (i.e., ≥60 minutes all 7 days), daily physical education (i.e., attended all 5 days), and playing on sports teams have all been associated with higher levels of feeling close to persons at school, which helps students engage in positive health behaviors and avoid many risk behaviors (*8*,*9*). Physical activity also has been identified as a protective factor for adolescents because it positively affects self-esteem, relationships, and academic achievement (*1*,*10*). Therefore, students who experience situations that make them feel unsafe might engage in physical activity as an outlet for coping with increased stress and anxiety (*11*).

Certain results observed in this report differed from those of a similar 2009 study, but both studies found positive and negative relations between violence-related behaviors and physical activity behaviors in high school students (*6*). For example, among male students who were bullied on school property, the 2009 study demonstrated a negative association with daily physical activity, while the results in this report demonstrate a negative association with physical education (*6*). One similar finding in both reports was a positive association among male students threatened or injured with a weapon on school property and sports team participation (*6*). In an analysis of 2019 YRBS data, adolescent girls who experienced sexual violence were found to have engaged in muscle-strengthening exercises more often; one possible reason is that students might turn to strength training as a means of protecting themselves from future acts of violence, as has been noted among anabolic steroid users who have experienced sexual violence (*12*,*13*). Alternatively, muscle-strengthening and playing sports might provide a positive outlet for students who have experienced stressful situations.

Physical activity behaviors that are contingent on school practice and policy (e.g., physical education class attendance), might have a different relation to student-level safety and violence experiences. For example, female students who witnessed community violence and male students who were bullied electronically were less likely to attend physical education class on all 5 days, indicating a different directional relation with these negative safety and violence experiences than the other physical activity behaviors. Future studies are needed to understand how school policy or other factors related to school policy might affect sex-specific associations between negative safety and violence experiences and physical education attendance.

The cross-sectional nature of these data prevents causal inference. For example, although physical activity is known to help reduce symptoms of anxiety and depression (*1*), it is unclear whether students turn to physical activity as a way of reducing anxiety and depression or if they are exposed to negative safety and violence experiences (e.g., unsafe environments, sports hazing, or rivalries) while participating in physical activity. Being bullied at school was positively associated with meeting the aerobic physical activity guideline and meeting both physical activity guidelines for female students, but not male students. Physical environments related to physical activity might play a role in these relationships; locker rooms, for instance, have been identified as places within school settings associated with violence and bullying, especially for female students (*14*). This finding emphasizes that safe physical environments are important for both promoting physical activity and preventing youth violence (https://www.cdc.gov/violence-prevention/media/pdf/resources-for-action/cv-prevention-resource-for-action_508.pdf) in spaces where physical activity occurs.

Because schools have a unique opportunity to help students participate in the amount of physical activity beneficial for overall health, negative safety and violence experiences associated with students’ physical activity behaviors need to be carefully considered. Understanding female and male students’ current physical activity behaviors in the context of negative safety and violence experiences could be important for physical education teachers, coaches, athletic directors, and school counselors. For example, there could be an opportunity within physical education classes, sports teams, and school spaces that promote physical activity to discuss ways to constructively process stress that students face when they feel unsafe. The identified associations between negative safety and violence experiences and more frequent physical activity behaviors also reinforce the need for physical activity infrastructure and programming in the school setting as a protective factor for the well-being of children and adolescents (*2*).

These findings demonstrate that more high school students who have been threatened at school played on a youth sports team than students without these negative experiences. As schools and communities work to increase the proportion of children and adolescents who participate on a sports team or take sports lessons after school or on weekends (an objective of Healthy People 2030; https://health.gov/healthypeople), students could substantially benefit from ensuring that sports offer a healthy and safe environment and outlet for coping with stress. These considerations can help keep participation safe for students while increasing opportunities for school-based physical activity that are implemented as part of a Comprehensive School Physical Activity Program.

## Limitations

General limitations for the 2023 YRBS are described in the overview report of this supplement (*7*). The findings in this report are subject to at least two additional limitations. First, negative safety and violence experiences and physical activity behaviors had various frames of reference (i.e., past 7 days, past 30 days, and past year), which might affect any resulting relation between the experiences and the physical activity behaviors. Second, individual socioeconomic status measures are known to be associated with physical activity opportunities but were not available and thus not accounted for in the analyses (*15*).

## Future Directions

Continuing to monitor trends in physical activity behaviors to promote health and well-being could be beneficial to students. Future studies could link YRBS data to school and place-based data sets to determine school and community characteristics that might act as barriers or facilitators to creating safe environments and encouraging physical activity behaviors. Linking these data sets would allow for investigating how physical activity behaviors differ by other demographic and socioeconomic characteristics to further examine health disparities. This findings in this report also suggest the need for further research to explore causal relations between negative safety and violence experiences and physical activity behaviors, identify pathways to understand how violence increases stress, and investigate whether physical activity serves as a moderator when students feel unsafe. Future studies could use multivariable modeling to consider the association between multiple negative safety and violence experiences with a single physical activity behavior, which could help identify the most salient negative experience related to engaging in a physical activity behavior. Future surveys could also consider asking students directly whether negative safety and violence experiences motivated them to participate in more or less of a specific physical activity behavior. In addition, it would be beneficial to examine the differences between physical activity behaviors that students engage in on school grounds compared with those that occur in nonschool settings. Whether female students and male students might be subject to negative safety and violence experiences for reasons related to gender norms and physical activity is unknown. These experiences might include female students being bullied for having a muscular physique from muscle-strengthening or male students being bullied electronically for being unathletic and not attending physical education classes. These findings might also guide professional development opportunities to ensure coaches, physical education teachers, and athletic directors have access to knowledge, tools, and resources related to identifying and supporting students who have experienced violence or bullying while also creating safe, supportive environments for physical activity.

## Conclusion

Although physical activity is a protective factor and beneficial to health and well-being, fewer than one in five students met both the aerobic and muscle-strengthening guidelines. In addition, only half of students participated in physical education and sports, which are opportunities that increase their physical activity. Contrary to what was expected, negative safety and violence experiences were mostly associated with students being more likely to engage in physical activity behaviors, with the exception of physical education class attendance. Understanding the potential protective effect of physical activity behaviors and the physical activity environments and situations in which students might encounter negative safety and violence experiences can inform physical activity infrastructure and programming offered in a school setting. Further exploring these associations can help public health and educational leaders develop more effective school-based interventions as well as continue to build practices that promote physical activity in schools through a Comprehensive School Physical Activity Program. In practice, this study could serve as a reference point for initiating dialogue about ways of ensuring implementation of safe, supportive, and inclusive school and physical activity environments that address bullying and violence. Physical education teachers can play a critical role for supporting physical activity participation within schools and creating safe environments for physical activity while addressing the development of skills across the psychomotor, cognitive, social, and affective domains of learning throughout a student's journey towards developing foundational motivation and self-efficacy for lifelong physical activity.

## Footnotes

* 45 C.F.R. part 46.114; 21 C.F.R. part 56.114.
